# Mitonuclear mismatch alters nuclear gene expression in naturally introgressed *Rhinolophus* bats

**DOI:** 10.1186/s12983-021-00424-x

**Published:** 2021-09-06

**Authors:** Yuting Ding, Wenli Chen, Qianqian Li, Stephen J. Rossiter, Xiuguang Mao

**Affiliations:** 1 Institute of Estuarine and Coastal Research, East China Normal University, Shanghai, 200062 China; 2 School of Ecological and Environmental Sciences, East China Normal University, Shanghai, 200062 China; 3 School of Biological and Chemical Sciences, Queen Mary University of London, London, E1 4NS UK; 4 Institute of Eco-Chongming (IEC), East China Normal University, Shanghai, 200062 China

**Keywords:** Mitonuclear discordance, Gene expression, RNA-seq, Introgression

## Abstract

**Background:**

Mitochondrial function involves the interplay between mitochondrial and nuclear genomes. Such mitonuclear interactions can be disrupted by the introgression of mitochondrial DNA between taxa or divergent populations. Previous studies of several model systems (e.g. *Drosophila*) indicate that the disruption of mitonuclear interactions, termed mitonuclear mismatch, can alter nuclear gene expression, yet few studies have focused on natural populations.

**Results:**

Here we study a naturally introgressed population in the secondary contact zone of two subspecies of the intermediate horseshoe bat (*Rhinolophus affinis*), in which individuals possess either mitonuclear matched or mismatched genotypes. We generated transcriptome data for six tissue types from five mitonuclear matched and five mismatched individuals. Our results revealed strong tissue-specific effects of mitonuclear mismatch on nuclear gene expression with the largest effect seen in pectoral muscle. Moreover, consistent with the hypothesis that genes associated with the response to oxidative stress may be upregulated in mitonuclear mismatched individuals, we identified several such gene candidates, including *DNASE1L3*, *GPx3* and *HSPB6* in muscle, and *ISG15* and *IFI6* in heart.

**Conclusion:**

Our study reveals how mitonuclear mismatch arising from introgression in natural populations is likely to have fitness consequences. Underlying the processes that maintain mitonuclear discordance is a step forward to understand the role of mitonuclear interactions in population divergence and speciation.

**Supplementary Information:**

The online version contains supplementary material available at 10.1186/s12983-021-00424-x.

## Background

Mitochondria are crucial to the fitness of an organism by regulating energy production in the cell. In particular, the oxidative phosphorylation (OXPHOS) pathway, necessitates epistatic interactions between proteins encoded by both mitochondrial and nuclear (mitonuclear) genomes [1, 2]. Because mitochondrial DNA (mtDNA) typically has a higher mutation rate than nuclear DNA [3], those nuclear genes that code for mitochondrial proteins (N-mt genes) evolve compensatory mutations in order to maintain compatibility of mitonuclear genomes [4, 5]. Selection for this mitonuclear compatibility leads to co-evolution of these two genomes, resulting in mitonuclear co-adaptation in different isolated populations [6–8].

The co-adaptation between mitochondrial and nuclear genomes can be disrupted in cases where formerly isolated populations come into secondary contact and undergo hybridization with introgression of mtDNA [9]. In particular, in cases of extensive introgression of mtDNA with little or no introgression of nuclear DNA (nDNA), the mitochondrial genome becomes mismatched with its nuclear genetic background [10], potentially resulting in reduced fitness due to mitonuclear conflicts and/or incompatibility [11–15]. Therefore, mitonuclear conflict caused by mitochondrial and nuclear DNA mismatch (mitonuclear mismatch) has been documented as an important driver of intrinsic reproductive isolation [16] and may play an important role in speciation [17, 18].

Mitonuclear mismatch has been observed in many naturally occurring populations [19–21] and also in humans [22], and is commonly detectable as mitonuclear discordance [23]. Studying natural individuals that have identical or similar nuclear genetic backgrounds but with markedly different mitochondrial variants can help to understand the nature of mtDNA-nDNA interactions and the evolutionary consequences of mitonuclear mismatch on population divergence. So far, few studies have directly investigated the impact of mitonuclear mismatch on fitness in natural populations (but see [10, 24]), largely because it is difficult to compare the fitness of individuals with different combinations of mitochondrial and nuclear genotypes. To tackle this limitation, we can use indirect ways of assessing possible fitness consequences, such as by examining transcriptional differences in multiple tissues between individuals with matched and mismatched mitonuclear genomes. Up to now, only a few studies have used comparative transcriptomics to investigate the effects of mitonuclear interactions on gene expression, and nearly all such studies focused on the model organism *Drosophila* [25–27]. Most of these studies indicated that mtDNA variation could alter nuclear gene expression, particularly when individuals with mismatched mitonuclear genomes are placed under stress (e.g. hypoxic conditions). As far as we know, few such studies have been performed in naturally introgressed populations [28, 29], which are critical to understand how individuals with mismatched mitonuclear genotypes have evolved to cope with mitochondrial dysfunction, such as reduced ATP production [30] and elevated oxidative damage [31, 32].

**Fig. 1:**
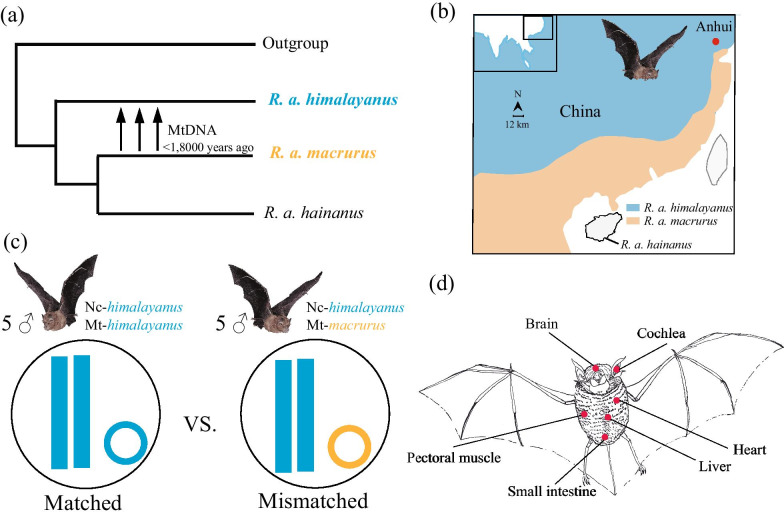
Experimental design to study the transcriptional effects of mitonuclear mismatch in *R. affinis*. **a** Phylogenetic relationships between the two hybridizing subspecies, *R. a. himalayanus* and *R. a. macrurus*. Extensive mtDNA introgression has occurred from *macrurus* to *himalayanus* in their secondary contact regions. **b** Geographic distribution of *macrurus* and *himalayanus* and the sampling locality of *himalayanus* in this study. **c** The study system includes five males of each *himalayanus* group, mitonuclear matched (both nuclear and mitochondrial genomes are from *himalayanus*) and mismatched (nuclear and mitochondrial genomes are from *himalayanus* and *macrurus*, respectively) groups. **d** We sampled six adult tissues (pectoral muscle, heart, brain, liver, cochlea, and small intestine) to assess transcriptional variations of nuclear genes between matched and mismatched groups

Bats are the only mammals to have evolved powered flight, and, for this reason, they have extremely high metabolic rates compared to other similar-sized mammals [33]. It is therefore likely that bats are unable to tolerate mitochondrial dysfunction caused by mitonuclear mismatches. Here we focus on the intermediate horseshoe bat (*Rhinolophus affinis*) which includes three recently diverged subspecies in China: two mainland subspecies (*R. a. himalayanus* and *R. a. macrurus*) and one island species from Hainan (*R. a. hainanus*) (Fig. 1a, see also [34, 35]). These three subspecies diverged less than one million years ago, and our previous phylogenetic studies of this species have indicated that *himalayanus* colonized Hainan to form *hainanus* during a period of glaciation, which then recolonized the mainland to form *macrurus* [34–36]. Extensive mitochondrial introgression (almost complete replacement of mitochondrial genome) with little or no nuclear introgression, appears to have occurred from *macrurus* to *himalayanus* in regions of secondary contact, following the recent population expansion of *macrurus* soon after the last glacial maximum (~ 18,000 years ago) (Fig. 1a, b, see also [34–37]). The discordant mitonuclear introgression between these two subspecies has resulted in mismatched mitonuclear genomes of *R. a. himalayanus* individuals, providing a unique model system in which to investigate the transcriptional impact of mitonuclear mismatch in naturally introgressed populations. Here we compare individuals with matched mitonuclear genotypes (both nuclear genome and mitogenome are from *R. a. himalayanus*) to ones with mismatched mitonuclear genotypes (nuclear genome is from *R. a. himalayanus* while mitogenome is from *R. a. macrurus*) (Fig. 1c).

Because mitochondria in mitonuclear mismatched individuals tend to generate higher levels of reactive oxygen species (ROS) [38, 39] and elevated oxidative damage [31, 32], we hypothesized that genes with roles in the protection of cells against oxidative stress would be significantly upregulated in mismatched individuals. To test this hypothesis, we assess transcriptional variations of nuclear genes in multiple adult tissues (Fig. 1d). Specifically, we examine differentially expressed genes (DEGs) between mitonuclear mismatched and matched individuals (also see [25–27]). These DEGs may be essential for cells or organisms to respond to the cellular stress caused by mitonuclear mismatch in natural conditions.

## Methods

### Sampling and experimental design

We previously found that many *Rhinolophus affinis himalayanus* individuals sampled from Anhui and Jiangxi provinces showed introgression of mtDNA from *R. a. macrurus* [37]. In this study we captured 10 adult males of *R. a. himalayanus* from one cave in Anhui province, China in 2019 (Fig. 1b). *R. a. himalayanus* is not an endangered species in the local region. Bats were euthanized by cervical dislocation. For each individual, we collected six tissues (pectoral muscle, heart, brain, liver, cochlea, and small intestine) with RNase-free tubes. We also collected the brain tissue from four *R. a. macrurus* individuals used in our previous study [40] in order to examine the sequence differences between the two subspecies (see below). Tissues were frozen immediately in liquid nitrogen and stored in a − 80 °C freezer.

To identify *himalayanus* individuals with or without introgression of *macrurus* mtDNA, we reconstructed phylogenetic relationships between these newly sampled individuals and previous ones from *macrurus* and *himalayanus*. For this, we sequenced the cytochrome b (cytb) gene from new samples and downloaded cytb sequences published previously from five *macrurus* and five *himalayanus* [36]. Primers and PCR profiles for amplification of cytb gene have been described previously [41]. MEGA 6 [42] was used to align all sequences and a Neighbor-Joining (NJ) tree was reconstructed with 1000 bootstraps. The NJ tree revealed that five of the new *himalayanus* individuals had mitochondrial haplotypes that clustered with *macrurus* while the remaining five individuals clustered with *himalayanus* (Additional file 1: Supporting Figure S1a). Thus, we separated these 10 new *himalayanus* individuals into two groups, one of five individuals with *macrurus* mtDNA (mismatched group, Nc-*himalayanus*:Mt-*macrurus*) and the other one of five individuals with *himalayanus* mtDNA (matched group, Nc-*himalayanus*:Mt-*himalayanus*) (Fig. 1c and Table 1).

**Table 1:** Details of 10 *R. a. himalayanus* individuals used for RNA-seq analyses. Among them, two individuals (Him-16 and Him-17) used for whole-genome resequencing analysis were shown with *

Sample ID	Groups	Description
Him-03	Nc-*himalayanus*::Mt-*macrurus*	*himalayanus* with *macrurus* mtDNA
Him-07	Nc-*himalayanus*::Mt-*macrurus*	*himalayanus* with *macrurus* mtDNA
Him-16*	Nc-*himalayanus*::Mt-*macrurus*	*himalayanus* with *macrurus* mtDNA
Him-18	Nc-*himalayanus*::Mt-*macrurus*	*himalayanus* with *macrurus* mtDNA
Him-24	Nc-*himalayanus*::Mt-*macrurus*	*himalayanus* with *macrurus* mtDNA
Him-08	Nc-*himalayanus*::Mt-*himalayanus*	*himalayanus* with *himalayanus* mtDNA
Him-12	Nc-*himalayanus*::Mt-*himalayanus*	*himalayanus* with *himalayanus* mtDNA
Him-17*	Nc-*himalayanus*::Mt-*himalayanus*	*himalayanus* with *himalayanus* mtDNA
Him-19	Nc-*himalayanus*::Mt-*himalayanus*	*himalayanus* with *himalayanus* mtDNA
Him-22	Nc-*himalayanus*::Mt-*himalayanus*	*himalayanus* with *himalayanus* mtDNA

### Transcriptome sequencing, genome resequencing and raw data trimming

For each tissue sample, we extracted total RNA extraction with TRIzol (Life Technologies Corp., Carlsbad, CA, USA) and conducted library constructions using Illunima’s TruSeq mRNA Standard library preparation kit. One library from the muscle tissue was discarded due to its low quality. All the remaining 63 libraries were quantified and qualified with Agilent 2100 Bioanalyzer and sequenced with Illumina HiSeq X Ten (paired-end 150 bp). Overall, we generated RNA-seq data for 59 *himalayanus* and four *macrurus* samples and obtained an average of 22 million reads per sample (Additional file 4: Table S1).

We generated ~ 30 Gb data with the whole-genome sequencing coverage of 15 × for each of the two individuals (Him-16 and Him-17) representing mismatched and matched groups, respectively (see below). Genomic DNA was extracted using muscle tissue with DNeasy kits (Qiagen) and was quantified using a Qubit 2.0 Fluorometer. DNA libraries were constructed using the NEB Next Ultra DNA Library Prep kit (New England Biosciences) and sequenced on the Illumina HiSeq X Ten (paired-end 150 bp).

Raw reads from each sample were trimmed with TRIMMOMATIC version 0.38 [43] using a sliding window of 4 bp with minimum average PHRED quality score of 20 and minimum reads length of 50 bp.

### Analysis of nuclear genome sequence

To characterize the nuclear genome difference between the two *himalayanus* groups, we first conducted an individual-based principal component analysis (PCA) with PLINK version-1.07 [44] based on SNPs datasets. For SNPs calling, we used RNA-seq reads of the brain tissue from all 10 *himalayanus* individuals and four *macrurus*. Briefly, we mapped filtered reads from each individual to the chromosome-level reference genome of *R. a. himalayanus* (G. Li, unpublished data) using BWA-MEM with default parameters [45]. SAMtools Version 1.9 [46] was used to generate sorted BAM file and to remove potential PCR duplicates. We used BCFtools Version 1.9 [47] to perform multisample SNPs calling. For each sample, we only kept high quality SNPs with the mapping quality (MQ) > 20, base quality (phred-scaled confidence) > 30 and sequence coverage (read depth) > 10. Second, based on SNP datasets we calculated the Weir and Cockerham estimator of Fst [48] between the two *himalayanus* groups using Vcftools v0.1.16 [49]. Because our main aim here is to estimate the general level of genetic differentiation between the two *himalayanus* groups, we applied a nonoverlapping window of 100 kb, and only windows with the minimum of 10 SNPs were used to calculate the weighted Fst values. Third, to further test for the genome similarity of the two *himalayanus* groups, we examined sequence differences at nuclear-encoded OXPHOS genes because their proteins show direct interactions with mitochondrial encoded proteins. We generated sequences of 68 OXPHOS genes [50]from each of the 14 individuals (10 *himalayanus* and 4 *macrurus*) (see details in Additional file 3: Supporting information and Additional file 9: Table S6). These sequences were then aligned using MEGA 6 and amino acid changes were calculated across the 14 individuals.

### Analysis of mitochondrial genome sequence

We previously generated a complete mitogenome for Him-17 (GenBank accession: MT845219) [51], belonging to matched group. To compare sequence differences of the complete mitogenome between mismatched and matched groups, we generated the complete mitogenome for Him-16 (mismatched group) using similar procedures as in Ding et al. [51] (see details in Additional file 3: Supporting information). Nucleotide differences between Him-16 and Him-17 were calculated for 37 mitochondrial genes and control region using MEGA.

To investigate whether sequence differences between Him-16 and Him-17 were fixed in each group, we generated the 13 mitochondrial protein-coding genes (PCGs) for the remaining eight *himalayanus* individuals and four *macrurus* individuals (see details in Additional file 3: Supporting information). All sequences were aligned using MEGA and amino acid changes across all 14 individuals were calculated for each of the 13 PCGs. Aligned sequences of concatenated 13 PCGs were used as the input to build a Maximum-likelihood (ML) tree using RaxML with GTRGAMMA model and bootstraps 1000 setting. *R. ferrumequinum* (GenBank accession: KT779432) was used as the outgroup.

To complement the above nuclear analyses, we also conducted PCA and sliding-window analyses based on SNPs dataset of mtDNA using similar procedures as above. SNPs were generated using the mitogenome of Him-17 as the reference. For the sliding-window Fst analysis, we used a nonoverlapping window size of 1 Kb.

### Differential expression analysis

For each tissue, filtered reads from each sample were mapped to the reference genome of *R. a. himalayanus* using Hisat2 [52] with default settings. Mapped reads were quantified using Featurecount [53]. We removed those lowly expressed transcripts with less than one CPM (counts per million) across samples. Read count matrices across samples were normalized in DESeq2 [54]. Prior to differential expression analysis (DE), we checked for potential outliers in each tissue using *PcaGrid* method [55] implemented in the *rrcov* R package with default parameters. Out of the 59 samples, one brain sample, two liver samples, one cochlea sample, and one intestine sample were identified as significant outliers and therefore were not used in DE analysis. Samples were renormalized after removal of outliers in each tissue. We identified differentially expressed genes (DEGs) between the two *R. a. himalayanus* groups in each tissue using DESeq2. A gene was considered as DEG using |log_2_ (fold change)|> 1 and *P* value < 0.05 after Benjamini and Hochberg adjustment for multiple tests (padj < 0.05) [56].

To examine the functional role of DEGs identified in each tissue, we performed enrichment analysis using Metascape (http://metascape.org) [57]. Significance was determined using Bonferroni corrected *p* value of < 0.05. We reduced redundancy of significant GO (Gene Ontology) terms using the REVIGO clustering algorithm (http://revigo.irb.hr/) [58] and the clustered GO terms were visualized using scatterplots with the semantic similarities of GO terms.

## Results

### Mitogenome analysis

We found a total of 504 nucleotide differences (2.98% of mitogenome) between the mitogenomes of Him-16 and Him-17, which represent mismatched and matched groups respectively (Additional file 2: Figure S2a and Additional file 5: Table S2). This overall high genetic differentiation across the whole mitogenome was also shown by a sliding-window analysis of Fst between the two groups (Additional file 2: Figure S2d). Among the 504 differences observed, 17 were non-synonymous, giving rise to 16 amino acid changes in mitochondrial protein-coding genes (PCGs) (Additional file 5: Table S2). Using RNA-seq data, we obtained sequences of all 13 PCGs from 10 *himalayanus* and 4 *macrurus* individuals. We observed 14 fixed amino acid changes between matched and mismatched groups (Additional file 6: Table S3). Thirteen of these were shared between mismatched group and *macrurus*, indicating the occurrence of introgression from *macrurus* to *himalayanus*. Consistent with this, the ML tree based on sequences of concatenated 13 PCGs and PCA based on SNPs from the whole mitogenome also strongly supported the classification of five mismatched individuals with *macrurus* (Additional file 1: Figure S1b, Additional file 2: S2b).

### Nuclear genome analysis

A further PCA based on SNPs using RNA-seq data classified all *himalayanus* individuals together and separated them from *macrurus* (Additional file 2: Figure S2c). Our sliding-window analysis of Fst across each chromosome also supported a low genetic differentiation between the two *himalayanus* groups with only six windows characterized by a Fst value of > 0.5 (Additional file 2: Figure S2e). Lastly, we did not detect fixed amino acid changes between the two *himalayanus* groups at 68 nuclear-encoded OXPHOS genes. Instead, we found eight fixed amino acid changes between *himalayanus* and *macrurus* occurring at seven genes (Additional file 7: Table S4).

### Differential expression analysis

**Fig. 2:**
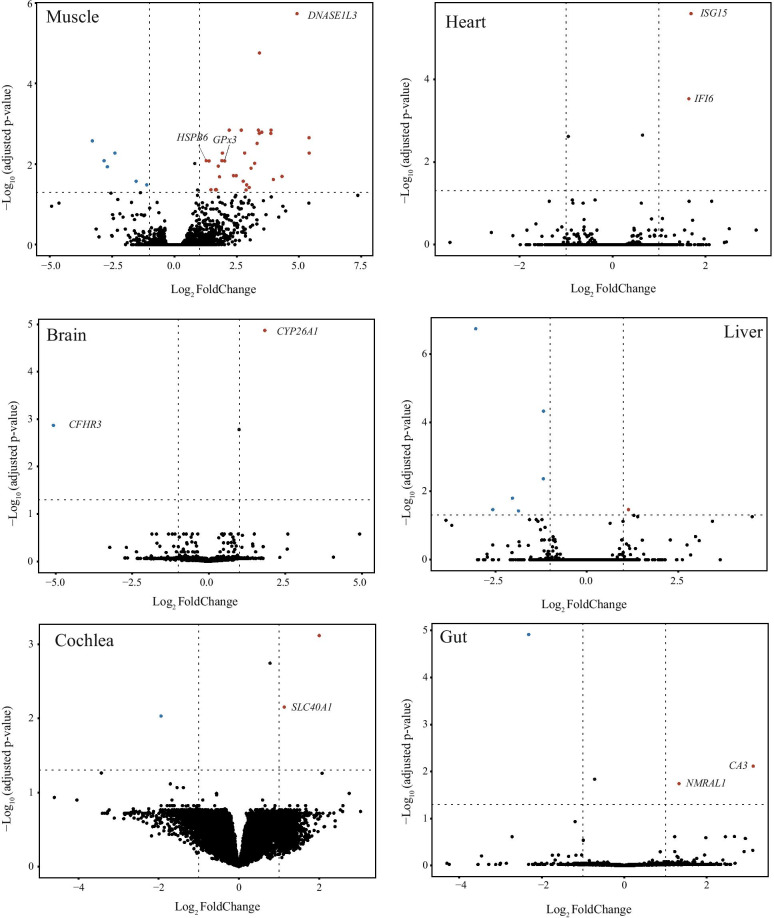
Volcano plots showing overall gene expression in each tissue between matched and mismatched groups with expression fold changes and adjusted P values for each gene. Differentially expressed genes that were discussed in the text were shown with their names

To investigate transcriptional response to mitonuclear mismatch in natural populations, we examine changes of gene expression between mitonuclear mismatched and matched groups (see Fig. 1c) across six adult tissues. Overall, we identified a small number of differentially expressed genes (DEGs) with a total of 60 DEGs across all six tissues. Among these six tissues, muscle showed the largest number with 43 DEGs, and the remaining tissues each showed fewer than 10 DEGs (Fig. 2 and Table 2). No DEGs were common to all six tissues.

**Table 2:** Differentially expressed genes (DEGs) identified in each tissue

DEGs	Log_2_CPM	Log_2_FC	*p*-adj	Gene description
*Pectoral muscle*				
DNASE1L3	1.42	6.63	8.90E−04	Deoxyribonuclease 1 like 3
CLNK	0.20	5.85	7.96E−03	Cytokine dependent hematopoietic cell linker
ACTC1	5.35	5.41	5.00E−03	Actin alpha cardiac muscle 1
TNNT2	0.58	5.40	2.39E−03	Troponin T2, cardiac type
CXCL10	1.89	4.92	2.47E−06	C-X-C motif chemokine ligand 10
SLAMF6	0.90	4.32	2.18 E−02	SLAM family member 6
IL21R	1.72	4.19	2.71E−02	Interleukin 21 receptor
VPREB3	1.09	3.97	2.58E−02	V-set pre-B cell surrogate light chain 3
CD3G	1.76	3.88	1.44E−03	CD3g molecule
XCL1	1.27	3.87	1.97E−03	X-C motif chemokine ligand 1
CD7	2.06	3.50	1.70E−03	CD7 molecule
WDFY4	3.50	3.41	2.12E−05	WDFY family member 4
IRF8	2.83	3.41	1.95E−03	Interferon regulatory factor 8
CPVL	3.52	3.37	1.44E−03	Carboxypeptidase vitellogenic like
MYL4	2.80	3.32	3.52E−03	Myosin light chain 4
RYR3	2.68	3.22	9.62E−03	Ryanodine receptor 3
LCK	3.16	3.07	1.32E−02	LCK proto-oncogene, Src family tyrosine kinase
PRUNE2	0.97	3.00	4.15E−02	Prune homolog 2 with BCH domain
XIRP2	7.30	2.90	2.96E−02	xin actin binding repeat containing 2
CD3E	2.67	2.87	4.46E−02	CD3e molecule
IL2RB	2.27	2.82	5.73E−03	interleukin 2 receptor subunit beta
LAT	1.52	2.76	2.71E−02	Linker for activation of T cells
MPEG1	3.08	2.68	1.44E−03	Macrophage expressed 1
ITM2A	2.31	2.48	2.03E−02	Integral membrane protein 2A
CTSW	2.13	2.37	2.03E−02	Cathepsin W
CILP	3.51	2.20	1.44E−03	Cartilage intermediate layer protein
GPX3	5.21	2.02	8.05E−03	Glutathione peroxidase 3
CD74	7.86	1.92	5.70E−03	CD74 molecule
ARHGAP22	1.81	1.92	7.96E−03	Rho GTPase activating protein 22
SYNPO2	5.17	1.90	7.96E−03	Synaptopodin 2
HLA-DRA	6.85	1.81	2.06E−02	Major histocompatibility complex, class II, DR alpha
POSTN	2.44	1.76	1.16E−02	Periostin
PLIN1	5.38	1.69	4.16E−02	Perilipin 1
MFAP5	2.23	1.64	4.22E−02	Microfibril associated protein 5
COL1A2	4.44	1.47	4.22E−02	Collagen type I alpha 2 chain
MAP1A	3.59	1.39	8.05E−03	Microtubule associated protein 1A
HSPB6	7.15	1.27	7.96E−03	Heat shock protein family B (small) member 6
SLC16A5	5.94	− 1.11	2.96E−02	Solute carrier family 16 member 5
CATSPER2	2.06	− 1.54	2.71E−02	Cation channel sperm associated 2
CSF3R	3.68	− 2.39	5.73E−03	Colony stimulating factor 3 receptor
CYP4F2	3.20	− 2.69	1.21E−02	Cytochrome P450 family 4 subfamily F member 2
IL1B	3.72	− 2.82	7.96E−03	Interleukin 1 beta
CCL8	2.16	− 3.30	2.83E−03	C–C motif chemokine ligand 8
*Heart*				
ISG15	3.31	1.69	2.67E−06	ISG15 ubiquitin like modifier
IFI6	5.41	1.64	3.03E−04	Interferon alpha inducible protein 6
*Brain*				
CYP26A1	5.46	1.83	1.35E−05	Cytochrome P450 family 26 subfamily A member 1
CFHR3	0.56	− 5.08	1.35E−03	Complement factor H related 3
*Liver*				
HLA-A	7.78	1.14	3.68E−02	HLA class I histocompatibility antigen, A alpha chain
SULT2A1	7.12	− 1.18	4.36E−05	Sulfotransferase family 2A member 1
ANKH	3.35	− 1.19	4.34E−03	ANKH inorganic pyrophosphate transport regulator
SPP1	5.18	− 1.87	3.99E−02	Secreted phosphoprotein 1
WFDC2	3.27	− 2.04	1.73E−02	WAP four-disulfide core domain 2
RGS4	0.72	− 2.57	3.73E−02	Regulator of G-protein signaling 4
TGM1	3.24	− 3.04	2.08E−07	Protein-glutamine gamma-glutamyltransferase K
*Cochlea*				
SCTR	2.97	2.00	8.05E−04	Secretin receptor
SLC40A1	4.64	1.13	7.06E−03	Solute carrier family 40 member 1
MYOC	7.71	− 1.94	8.99E−03	Myocilin
*Small intestine*				
CA3	3.63	3.13	8.11E−03	Carbonic anhydrase 3
NMRAL1	2.36	1.33	2.11E−02	NmrA-like family domain containing 1
PCDH8	3.36	− 2.32	1.38E−05	Protocadherin 8

Positive and negative values indicate that genes are upregulated and downregulated in mismatched group, respectively. CPM and FC indicate counts per million and fold change, respectively

**Fig. 3:**
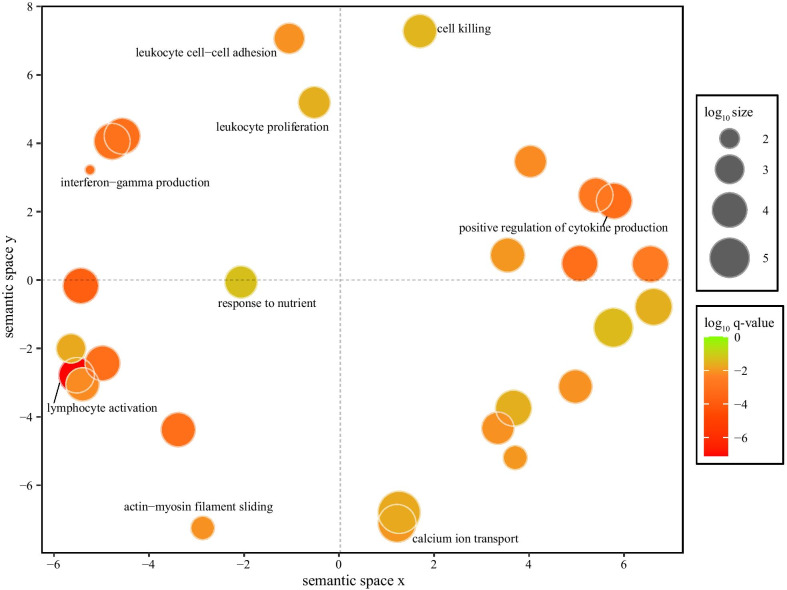
Multidimensional scaling plot showing shared GO terms enriched for differentially expressed genes identified in muscle. Clustering was conducted based on semantic similarity of GO terms. The colour and size of circles correspond to the q-value calculated by Metascape and the frequency of the GO term in GO annotation database, respectively. Only the highly shared GO terms are shown with names

Functional enrichment analysis on DEGs in muscle revealed 103 significant GO terms (Additional file 8: Table S5). After reducing redundancy, we obtained 27 GO terms and most of them are related to immune response including cell killing, positive regulation of cytokine production, lymphocyte activation, leukocyte proliferation, and interferon-gamma production (Fig. 3). We also found terms associated with actin-myosin filament sliding and calcium ion transport (Fig. 3 and Additional file 8: Table S5). In contrast to muscle, we did not find any significant GO terms among DEGs identified in the other five tissues examined.

## Discussion

Mitochondrial function relies on epistatic interactions among mitochondrial and nuclear genes. This mitonuclear interaction leads to co-adapted mitonuclear genotypes in isolated populations or lineages. In cases where allopatric populations show high levels of divergence in their mitogenomes, then mtDNA introgression on secondary contact can disrupt the co-adapted mitonuclear genotypes and lead to mitonuclear mismatches in hybrid populations [7, 8]. It is known that mitonuclear mismatch can reduce the efficiency of ATP production in the OXPHOS process and promote higher level of ROS production [38, 39], with potential negative fitness consequences [27, 59]. Yet while introgression of mtDNA has been commonly documented [60, 61], very few studies have examined the effects of this mtDNA introgression (i.e. mitonuclear mismatch) on nuclear gene expression in natural conditions.

In this study, we conducted, to our knowledge, the first assessment of the transcriptional effects of mismatched mitonuclear genotype within species across multiple adult tissues. We sampled one *R. a. himalayanus* population from the secondary contact region of *R. a. himalayanus* and *R. a. macrurus*, and found that half of all sampled *himalayanus* individuals exhibited introgression of mtDNA from *macrurus* (Additional file 1: Figure S1, Additional file 2: S2a and Additional file 2: S2e, Additional file 5: Table S2). This introgression of mtDNA was clear in phylogenetic analyses and in our PCA (Additional file 2: Figure S2c), with several fixed amino acid changes at mitochondrial protein coding genes (Additional file 6: Table S3) observed among the two *himalayanus* groups and *macrurus* (see also [35, 37]). Next we examine the nuclear divergence between the two *himalayanus* groups. The results of the PCA and sliding-window Fst analysis supported the overall similarity across the nuclear genome between these two groups (Additional file 2: Figure S2c and Additional file 2: Figure S2e) and our recent study based on a wider range of sampling also indicated that no nuclear DNA introgression had occurred between the two subspecies following mtDNA introgression [37].

Currently, more work is needed to determine the exact evolutionary forces underlying the introgression of mtDNA, alongside little or no trace of introgression of ncDNA (see a recent example in chipmunks [62]). One possible explanation is that the mitogenome confers an adaptive advantage (e.g. [63]). However, while signatures of positive selection have been detected in the mitogenome of *R. affinis*, demographic explanations could not be ruled out completely [37]. Alternatively, the mtDNA introgression might simply be a signal that has been left behind following hybridization, whereas ncDNA introgression was eroded via recombination following backcrossing [60]. In the future whole-genome resequencing data from multiple individuals will be needed to assess the level of nuclear introgression between the two mainland subspecies. In addition, whole-genome data can also be used to test for other mechanisms, such as demographic effects and selection against hybrids [18]. Nevertheless, the current findings provide strong support that the sampled *R. a. himalayanus* population includes two groups: one is a mitonuclear matched group whose mitogenome and nuclear genome are both from *himalayanus* (Nc-*himalayanus*:Mt-*himalayanus*), and the other is a mismatched group whose mitogenome is from *macrurus* but whose nuclear genome is from *himalayanus* (Nc-*himalayanus*:Mt-*macrurus*) (Fig. 1c).

Previously, the effects of mitonuclear mismatch on nuclear gene expression have been studied in *Drosophila* from RNA-seq data obtained from the whole organism [27]. In contrast, we examined transcriptional variation across multiple tissue types, and found clear tissue-specific effects of mismatched mitonuclear genotype. Specifically, mitonuclear mismatch showed a larger effect in pectoral muscle than in other tissues, with 43 DEGs identified in muscle but fewer than 10 in the other tissues, a pattern that is consistent with the fact that muscle usually requires more energy than other tissues for animals, which is likely to be especially true of volant species such as bats. An alternative explanation for why muscle shows greater number of DEGs than other tissues is that bulk RNA-seq, applied in this study, may have a larger effect on the identification of DEGs in heterogeneous tissues (e.g. brain and cochlear) than in homogeneous tissue (muscle). In the future, the application of single-cell transcriptomics approaches [64] might be informative in testing this possibility.

Although mtDNA from mismatched and matched groups shows only 14 fixed amino acid changes in protein-coding genes, our results demonstrate that this level of mtDNA difference still can likely have profound impacts on nuclear gene expression. In *Drosophila*, Mossman et al. [27] examined four different mtDNA haplotypes with 10 to 95 amino acid changes to assess the effects of mtDNA variation on nuclear gene expression, and identified similar numbers of DEGs in male comparisons. On the other hand, work on killifish revealed that only three nuclear genes were significantly differentially expressed between different mitochondrial genotypes in skeletal muscle [29]. However, because in this latter study the differences among mitochondrial genotypes (e.g. the number of amino acid changes or total mutations across the mitogenome) were not reported, the results cannot be compared directly to our findings, or to those of Mossman et al. [27]. Thus to draw general conclusions about the effects of different levels of divergent mitochondrial genotypes in natural populations, additional comparative transcriptomic studies of multiple tissues are needed.

We identified several significantly upregulated genes in mismatched individuals, the majority of which are associated with protection against the oxidative damage putatively caused by inefficiency of the OXPHOS pathway in the mitochondria. These genes may play essential roles for organisms in response to cellular stress in nature, and below we discuss some of these in each tissue specifically.

Most differentially expressed genes (DEGs) in muscle (37 of 43) showed upregulation in the mismatched group (Table 2). Among these, the highest expression was seen in *DNASE1L3* (deoxyribonuclease 1 like 3). The loss of this gene has been linked to aberrations in the fragmentation of DNA molecules [65] and the formation of anti-DNA antibodies and autoimmunity in mice and human [66]. It has been suggested that *DNASE1L3* could be activated by mitochondrial disruption [67]. We also found very high upregulation (4x) in the mismatched group of *GPx3* (glutathione peroxidase 3). Its encoded protein is a member of a family of antioxidant enzymes that is critical for the antioxidant effect of the peroxisome proliferator-activated receptor γ in response to oxidative stress in skeletal muscle cells [68]. The gene *HSPB6* encoded small heat shock protein HSPB6 which is most highly expressed in different types of muscle [69] and can be induced by oxidative stress [70]. Our functional enrichment analyses of DEGs in muscle revealed that a majority of DEGs are related to immune response, with other important terms associated with muscle contraction and calcium ion transport. Indeed, one term (GO:0033275) included three DEGs (*ACTC1*, *MYL4*, and *TNNT2*) with essential roles in muscle function, the former of which has also been shown to be overexpressed in mitochondrial myopathy due to mitochondrial respiratory chain deficiency [71]. Another important GO term (GO:0006816) includes six DEG, of which *CXCL10* showed almost 32-fold higher expression in the mismatched group compared to matched group; this gene was previously shown to be important in mitochondrial dysfunction and cellular apoptosis [72].

Although DEGs from other tissues did not reveal any significant functional enrichment based on GO terms, some of these loci nevertheless have known roles in protecting against mitochondrial dysfunction. Specifically, among the two DEGs from heart that were upregulated in the mismatched group, *ISG15* (interferon-stimulated gene 15) encodes a protein that is strongly associated with antiviral immune response [73] as well as regulation of mitochondrial OXPHOS and mitophagy processes during viral infection [74]. Recently, ISG15 has also been shown to play key roles in genome stability as a sensor of the DNA damage response and its expression is closely related to p53-mediated cellular processes [75, 76]. The second gene, *IFI6* (interferon alpha inducible protein 6), is also one of the interferon-stimulated genes, and encodes a mitochondrial localized protein. IFI6 might be connected with cellular ATP production and OXPHOS efficiency, by directly promoting mitochondrial supercomplex assembly [77]. We also found upregulation in the brain of the mismatched group, with an almost four-fold change in *CYP26A1*, which encodes cytochrome P450, a protein that has been shown to suppress oxidative stress-mediated apoptosis [78]. One upregulated gene in the cochlea of the mismatched group, *SLC40A1*, encodes an iron transporter and can regulate cell oxidative stresses [79]. Among two genes (*CA3* and *NMRAL1*) showing upregulation in the small intestine of the mismatched group, *CA3* encodes carbonic anhydrase 3, which functions as an antioxidant and has been proposed to have protective roles against oxidative damage [80], while NMRAL1 (also called HSCARG) acts as a cellular redox sensor and can regulate DNA damage response caused by severe oxidative stress [81]. Finally, in contrast to other tissues, almost all DEGs identified in the liver are downregulated in the mismatched group, although it is unclear whether these genes function in oxidative stress.

## Conclusions and future work

In our study system, we compared bats with near-identical nuclear genetic backgrounds but with contrasting histories of mtDNA introgression, and found significant and tissue-specific effects of mitonuclear mismatch on nuclear gene expression. Several genes upregulated in mismatched individuals encode proteins with known roles in responding to oxidative stress, making these potentially important candidates for future studies, including those of mitochondrial replacement therapy in human oocytes, a method used to treat mitochondrial diseases [82].

Because our current samples are all males, a remaining question that needs to be addressed is whether mitonuclear mismatches have similar effects on nuclear gene expression in females as they do in males. Previous studies aiming to answer this question have drawn different conclusions; some have indicated that mitochondrial variation have major effects on nuclear gene expression in males but little effects in females [83], a result that can be explained by the mother’s curse hypothesis, supporting a sex-specific selective sieve in mtDNA [83, 84]. However, other studies have demonstrated contradictory results, with smaller impacts of mtDNA polymorphism in males than in females [26, 85]. To address the sexual difference of mitonuclear mismatch effects on gene expression, more work needs to be performed on a wider range of animals.

When confirming the similarity of nuclear background in the two *himalayanus* groups, we focused on those nuclear-encoded OXPHOS genes because proteins encoded by these genes show direct interactions with mitochondrial encoded proteins. However, no fixed amino acid changes at these genes were found between the two *himalayanus* groups, indicating that nuclear-encoded mitochondrial genes (N-mt genes) might not have co-introgressed with mtDNA from *macrurus* to the mismatched group. Co-introgression of N-mt genes (mitonuclear co-introgression) has been considered as the most efficient way to overcome mitonuclear mismatches caused by mtDNA introgression [18], however, very few cases of mitonuclear co-introgression have been documented in natural populations [21, 86]. Recent studies have shown that co-introgression of N-mt genes may occur beyond those nuclear-encoded OXPHOS genes [2]. Further whole-genome resequencing data are needed to assess the extent of nuclear elements of *macrurus* in the genomes of mismatched individuals of *himalayanus*. In the future we also hope to investigate evolution of the nuclear genome under the strong selective force of foreign mtDNA by comparing genomic signature of differentiation between matched and mismatched *himalayanus* individuals [9, 87].

Mitonuclear mismatch is commonly detectable as mitonuclear discordance, and numerous studies have focused on understanding the processes that cause such discordance, such as incomplete lineage sorting or introgression. However, since mitonuclear discordance is likely an important intermediate state during the speciation, more work is needed to investigate the consequences of this discordance on survival and/or fitness of organism. Such studies could improve current understanding of the processes or factors that maintain mitonuclear discordance in natural populations [23]. While directly assessing the fitness consequences of mitonuclear discordance remains challenging for most wild animals (but see [10]), especially for those with long generation times, we show that studying the effects of mitonuclear genome mismatches on gene expression is more tractable (see also [26, 27, 85, 88]). Such approaches offer promising insights into the evolutionary changes and roles of natural selection in the process of adaptation to cellular stress caused by mitonuclear discordance in nature.

## Supplementary Information

**Additional file 1**. Phylogenetic relationships among samples of* R. a. himalayanus* and* R. a. macrurus* based on mtDNA.

**Additional file 2**. Sequence differences of mitogenome and nuclear genome between mitonuclear matched and mismatched individuals of* R. a. himalayanus*.

**Additional file 3**. Supporting Information for “Mitonuclear mismatch alters nuclear gene expression in naturally introgressed Rhinolophus bats”.

**Additional file 4: Table S1**. Detailed sequencing information of RNA-seq data and alignment rate to the reference genome for each sample.

**Additional file 5: Table S2**. Detailed differences in each position of the whole mitogenome between mitonuclear matched (Him-17) and mismatched (Him-16) individuals generated by whole-genome resequencing.

**Additional file 6: Table S3**. Fourteen fixed amino acid changes between matched group (Nc-*himalayanus*:Mt-*himalayanus*) and either mismatched group (Nc-*himalayanus*:Mt-*macrurus*) or *macrurus* at mitochondrial protein coding genes.

**Additional file 7: Table S4**. Fixed amino acid changes between two himalayanus groups (Nc-*himalayanus*:Mt-*himalayanus* and Nc-*himalayanus*:Mt-*macrurus*) and *macrurus* at nuclear-encoded OXPHOS genes.

**Additional file 8: Table S5**. Significant GO terms enriched on differentially expressed genes (DEGs) identified in pectoral muscle. GO terms named in Figure 3 are shown in bold.

**Additional file 9: Table S6**. List of 77 nuclear-encoded OXPHOS genes used in Shen et al. (2010).

## Data Availability

All raw sequencing reads have deposited to NCBI’s Sequence Read Archive database (SRA) (BioProject accession numbers: PRJNA727985; PRJNA740060; PRJNA740148). The datasets used and/or analysed during the current study are available from the corresponding author on reasonable request. All data generated or analysed during this study are included in this published article and its supplementary information files.

## Abbreviations

CPM: Counts per million
Cytb: Cytochrome b
DE: Differential expression
DEGs: Differentially expressed genes
DNASE1L3: Deoxyribonuclease 1 like 3
GO: Gene Ontology
GPx3: Glutathione peroxidase 3
IFI6: Interferon alpha inducible protein 6
ISG15: Interferon-stimulated gene 15
Log_2_FC: Log_2_ transformed fold change
ML: Maximum-likelihood
MQ: Mapping quality
mtDNA: Mitochondrial DNA
nDNA: Nuclear DNA
NJ: Neighbor-Joining
N-mt genes: Nuclear-encoded mitochondrial genes
OXPHOS: Oxidative phosphorylation
PCA: Principal component analysis
PCGs: Protein-coding genes
ROS: Reactive oxygen species
SRA: Sequence Read Archive database

## References

[1] Wolff JN, Ladoukakis ED, Enríquez JA, Dowling DK (2014). Mitonuclear interactions: evolutionary consequences over multiple biological scales. Philos Trans R Soc Lond B Biol Sci.

[2] Hill GE (2019). Mitonuclear ecology.

[3] Saccone C, Lanave C, De Grassi A (2006). Metazoan OXPHOS gene families: evolutionary forces at the level of mitochondrial and nuclear genomes. BBA-Bioenergetics.

[4] Hill GE (2020). Mitonuclear compensatory coevolution. Trends Genet.

[5] Bar-Yaacov D, Blumberg A, Mishmar D (2012). Mitochondrial-nuclear co-evolution and its effects on OXPHOS activity and regulation. Biochim Biophys Acta.

[6] Rand DM, Haney RA, Fry AJ (2004). Cytonuclear coevolution: the genomics of cooperation. Trends Ecol Evol.

[7] Dowling DK, Friberg U, Lindell J (2008). Evolutionary implications of non-neutral mitochondrial genetic variation. Trends Ecol Evol.

[8] Barreto FS, Watson ET, Lima TG, Willett CS, Edmands S, Li W (2018). Genomic signatures of mitonuclear coevolution across populations of *Tigriopus californicus*. Nat Ecol Evol.

[9] Healy TM, Burton RS (2020). Strong selective effects of mitochondrial DNA on the nuclear genome. Proc Natl Acad Sci U S A.

[10] Rank NE, Mardulyn P, Heidl SJ, Roberts KT, Zavala NA, Smiley JT (2020). Mitonuclear mismatch alters performance and reproductive success in naturally introgressed populations of a montane leaf beetle. Evolution.

[11] Burton RS, Pereira RJ, Barreto FS (2013). Cytonuclear genomic interactions and hybrid breakdown. Annu Rev Ecol Evol Syst.

[12] Burton RS, Barreto FS (2012). A disproportionate role for mtDNA in Dobzhansky–Muller incompatibilities?. Mol Ecol.

[13] Ma H, Gutierrez NM, Morey R, Van Dyken C, Kang E, Hayama T (2016). Incompatibility between nuclear and mitochondrial genomes contributes to an interspecies reproductive barrier. Cell Metab.

[14] Dobelmann J, Alexander A, Baty JW, Gemmell NJ, Gruber MAM, Quinn O (2019). The association between mitochondrial genetic variation and reduced colony fitness in an invasive wasp. Mol Ecol.

[15] Lee HY, Chou JY, Cheong L, Chang NH, Yang SY, Leu JY (2008). Incompatibility of nuclear and mitochondrial genomes causes hybrid sterility between two yeast species. Cell.

[16] Crespi B, Nosil P (2013). Conflictual speciation: species formation via genomic conflict. Trends Ecol Evol.

[17] Hill GE (2016). Mitonuclear coevolution as the genesis of speciation and the mitochondrial DNA barcode gap. Ecol Evol.

[18] Sloan DB, Havird JC, Sharbrough J (2017). The on-again, off-again relationship between mitochondrial genomes and species boundaries. Mol Ecol.

[19] Bar-Yaacov D, Hadjivasiliou Z, Levin L, Barshad G, Zarivach R, Bouskila A (2015). Mitochondrial involvement in vertebrate speciation? The case of mitonuclear genetic divergence in chameleons. Genome Biol Evol.

[20] Baris TZ, Wagner DN, Dayan DI, Du X, Blier PU, Pichaud N (2017). Evolved genetic and phenotypic differences due to mitochondrial–nuclear interactions. PLoS Genet.

[21] Morales HE, Pavlova A, Amos N, Major R, Kilian A, Greening C (2018). Concordant divergence of mitogenomes and a mitonuclear gene cluster in bird lineages inhabiting different climates. Nat Ecol Evol.

[22] Zaidi AA, Makova KD (2019). Investigating mitonuclear interactions in human admixed populations. Nat Ecol Evol.

[23] Streicher JW, Day JJ (2020). The toad's warts: discordance creates bumpy expectations of mitochondrial-nuclear evolution between species. Mol Ecol.

[24] Lee-Yaw JA, Jacobs CGC, Irwin DE (2014). Individual performance in relation to cytonuclear discordance in a northern contact zone between long-toed salamander (*Ambystoma macrodactylum*) lineages. Mol Ecol.

[25] Mossman JA, Biancani LM, Zhu CT, Rand DM (2016). Mitonuclear epistasis for development time and its modification by diet in *Drosophila*. Genetics.

[26] Mossman JA, Tross JG, Li N, Wu Z, Rand DM (2016). Mitochondrial-nuclear interactions mediate sex-specific transcriptional profiles in *Drosophila*. Genetics.

[27] Mossman JA, Ge JY, Navarro F, Rand DM (2019). Mitochondrial DNA fitness depends on nuclear genetic background in *Drosophila*. G3 Genes Genom Genet.

[28] Flight PA, Nacci D, Champlin D, Whitehead A, Rand DM (2011). The effects of mitochondrial genotype on hypoxic survival and gene expression in a hybrid population of the killifish, *Fundulus heteroclitus*. Mol Ecol.

[29] Healy TM, Bryant HJ, Schulte PM (2017). Mitochondrial genotype and phenotypic plasticity of gene expression in response to cold acclimation in killifish. Mol Ecol.

[30] Meiklejohn CD, Holmbeck MA, Siddiq MA, Abt DN, Rand DM, Montooth KL (2013). An incompatibility between a mitochondrial tRNA and its nuclear-encoded tRNA synthetase compromises development and fitness in *Drosophila*. PLoS Genet.

[31] Barreto FS, Burton RS (2013). Evidence for compensatory evolution of ribosomal proteins in response to rapid divergence of mitochondrial rRNA. Mol Biol Evol.

[32] Immonen E, Rönn J, Watson C, Berger D, Arnqvist G (2016). Complex mitonuclear interactions and metabolic costs of mating in male seed beetles. J Evol Biol.

[33] Thomas SP, Suthers RA (1972). The physiology and energetics of bat flight. J Exp Biol.

[34] Mao XG, Zhu GJ, Zhang SY, Rossiter SJ (2010). Pleistocene climatic cycling drives intra-specific diversification in the intermediate horseshoe bat (*Rhinolophus affinis*) in Southern China. Mol Ecol.

[35] Mao X, He G, Hua P, Jones G, Zhang S, Rossiter SJ (2013). Historical introgression and the persistence of ghost alleles in the intermediate horseshoe bat (*Rhinolophus affinis*). Mol Ecol.

[36] Mao X, Zhu G, Zhang L, Zhang S, Rossiter SJ (2014). Differential introgression among loci across a hybrid zone of the intermediate horseshoe bat (*Rhinolophus affinis*). BMC Evol Biol.

[37] Mao XG, Rossiter SJ (2020). Genome-wide data reveal discordant mitonuclear introgression in the intermediate horseshoe bat (*Rhinolophus affinis*). Mol Phylogenet Evol.

[38] Rand DM, Mossman JA, Zhu L, Biancani LM, Ge JY (2018). Mitonuclear epistasis, genotype-by-environment interactions, and personalized genomics of complex traits in *Drosophila*. IUBMB Life.

[39] Cocco T, Sgobbo P, Clemente M, Lopriore B, Grattagliano I, Di Paola M (2005). Tissue-specific changes of mitochondrial functions in aged rats: effect of a long-term dietary treatment with N-acetylcysteine. Free Radical Biol Med.

[40] Sun HJ, Chen WL, Wang JY, Zhang LB, Rossiter SJ, Mao XG (1934). Echolocation call frequency variation in horseshoe bats: molecular basis revealed by comparative transcriptomics. P Roy Soc B-Biol Sci.

[41] Mao X, Zhang J, Zhang S, Rossiter SJ (2010). Historical male-mediated introgression in horseshoe bats revealed by multilocus DNA sequence data. Mol Ecol.

[42] Tamura K, Stecher G, Peterson D, Filipski A, Kumar S (2013). MEGA6: molecular evolutionary genetics analysis version 6.0. Mol Biol Evol.

[43] Bolger AM, Lohse M, Usadel B (2014). Trimmomatic: a flexible trimmer for Illumina sequence data. Bioinformatics.

[44] Purcell S, Neale B, Todd-Brown K, Thomas L, Ferreira MA, Bender D (2007). PLINK: a toolset for whole-genome association and population-based linkage analysis. Am J Hum Genet.

[45] Li H, Durbin R (2009). Fast and accurate short read alignment with burrows-wheeler transform. Bioinformatics.

[46] Li H, Handsaker B, Wysoker A, Fennell T, Ruan J, Homer N (2009). The sequence alignment/map format and SAMtools. Bioinformatics.

[47] Li H (2011). A statistical framework for SNP calling, mutation discovery, association mapping and population genetical parameter estimation from sequencing data. Bioinformatics.

[48] Weir BS, Cockerham CC (1984). Estimating F-statistics for the analysis of population structure. Evolution.

[49] Danecek P, Auton A, Abecasis G, Albers CA, Banks E, DePristo MA (2011). The variant call format and VCFtools. Bioinformatics.

[50] Shen YY, Liang L, Zhu ZH, Zhou WP, Irwin DM, Zhang YP (2010). Adaptive evolution of energy metabolism genes and the origin of flight in bats. Proceed Nat Acad Sci.

[51] Ding Y, Chen W, Mao X (2021). The complete mitochondrial genome of *Rhinolophus affinis himalayanus*. Mitochondrial DNA B.

[52] Kim D, Langmead B, Salzberg SL (2015). HISAT: a fast spliced aligner with low memory requirements. Nat Methods.

[53] Liao Y, Smyth GK, Shi W (2014). FeatureCounts: an efficient general purpose program for assigning sequence reads to genomic features. Bioinformatics.

[54] Love MI, Huber W, Anders S (2014). Moderated estimation of fold change and dispersion for RNA-seq data with DESeq2. Genome Biol.

[55] Croux C, Filzmoserb P, Oliveirac MR (2007). Algorithms for projection-pursuit robust principal component analysis. Chemometr Intell Lab.

[56] Benjamini Y, Hochberg Y (1995). Controlling the false discovery rate: a practical and powerful approach to multiple testing. J R Stat Soc B.

[57] Zhou Y, Zhou B, Pache L, Chang M, Khodabakhshi AH, Tanaseichuk O (2019). Metascape provides a biologist-oriented resource for the analysis of systems-level datasets. Nat Commun.

[58] Supek F, Bošnjak M, Škunca N, Šmuc T (2011). REVIGO summarizes and visualizes long lists of gene ontology terms. PLoS ONE.

[59] Chang CC, Rodriguez J, Ross J (2015). Mitochondrial–nuclear epistasis impacts fitness and mitochondrial physiology of interpopulation *Caenorhabditis briggsae* hybrids. G3 Genes Genom Genet.

[60] Toews DPL, Brelsford A (2012). The biogeography of mitochondrial and nuclear discordance in animals. Mol Ecol.

[61] Hill GE (2019). Reconciling the mitonuclear compatibility species concept with rampant mitochondrial introgression. Integr Comp Biol.

[62] Sarver BA, Herrera ND, Sneddon D, Hunter SS, Settles ML, Kronenberg Z, Demboski JR, Good JM, Sullivan J (2021). Diversification, introgression, and rampant cytonuclear discordance in Rocky Mountains Chipmunks (Sciuridae: *Tamias*). Syst Biol.

[63] Melo-Ferreira J, Vilela J, Fonseca MM, da Fonseca RR, Boursot P, Alves PC (2014). The elusive nature of adaptive mitochondrial DNA evolution of an arctic lineage prone to frequent introgression. Genome Biol Evol.

[64] Kulkarni A, Anderson AG, Merullo DP, Konopka G (2019). Beyond bulk: a review of single cell transcriptomics methodologies and applications. Curr Opin Biotechnol.

[65] Serpas L, Chan RWY, Jiang P, Ni M, Sun K, Rashidfarrokhi A (2019). Dnase1l3 deletion causes aberrations in length and end-motif frequencies in plasma DNA. Proc Natl Acad Sci U S A.

[66] Sisirak V, Sally B, D'Agati V, Martinez-Ortiz W, Özçakar ZB, David J (2016). Digestion of chromatin in apoptotic cell microparticles prevents autoimmunity. Cell.

[67] Shi G, Abbott KN, Wu W, Salter RD, Keyel PA (2017). Dnase1L3 regulates inflammasome-dependent cytokine secretion. Front Immunol.

[68] Chung SS, Kim M, Youn BS, Lee NS, Park JW, Lee IK (2009). Glutathione peroxidase 3 mediates the antioxidant effect of peroxisome proliferator-activated receptor γ in human skeletal muscle cells. Mol Cell Biol.

[69] Salinthone S, Tyagi M, Gerthoffer WT (2008). Small heat shock proteins in smooth muscle. Pharmacol Ther.

[70] Wang X, Zhao T, Huang W, Wang T, Qian J, Xu M (2009). Hsp20-engineered mesenchymal stem cells are resistant to oxidative stress via enhanced activation of Akt and increased secretion of growth factors. Stem Cells.

[71] Tyynismaa H, Carroll CJ, Raimundo N, Ahola-Erkkilä S, Wenz T, Ruhanen H (2010). Mitochondrial myopathy induces a starvation-like response. Hum Mol Genet.

[72] Singh L, Arora SK, Bakshi DK, Majumdar S, Wig JD (2010). Potential role of CXCL10 in the induction of cell injury and mitochondrial dysfunction. Int J Exp Pathol.

[73] Perng YC, Lenschow DJ (2018). ISG15 in antiviral immunity and beyond. Nat Rev Microbiol.

[74] Baldanta S, Fernández-Escobar M, Acín-Perez R, Albert M, Camafeita E, Jorge I (2017). *ISG15* governs mitochondrial function in macrophages following vaccinia virus infection. PLoS Pathog.

[75] Tigano M, Vargas DC, Tremblay-Belzile S, Fu Y, Sfeir A (2021). Nuclear sensing of breaks in mitochondrial DNA enhances immune surveillance. Nature.

[76] Sandy Z, da Costa IC, Schmidt CK (2020). More than meets the ISG15: emerging roles in the DNA damage response and beyond. Biomolecules.

[77] Liu Z, Gu S, Lu T, Wu K, Li L, Dong C (2020). IFI6 depletion inhibits esophageal squamous cell carcinoma progression through reactive oxygen species accumulation via mitochondrial dysfunction and endoplasmic reticulum stress. J Exp Clin Cancer Res.

[78] Osanai M, Sawada N, Lee GH (2010). Oncogenic and cell survival properties of the retinoic acid metabolizing enzyme, CYP26A1. Oncogene.

[79] Skjørringe T, Møller LB, Moos T (2012). Impairment of interrelated iron- and copper homeostatic mechanisms in brain contributes to the pathogenesis of neurodegenerative disorders. Front Pharmacol.

[80] Monti DM, De Simone G, Langella E, Supuran CT, Di Fiore A, Monti SM (2017). Insights into the role of reactive sulfhydryl groups of Carbonic Anhydrase III and VII during oxidative damage. J Enzyme Inhib Med Chem.

[81] Zang W, Zheng X (2020). Structure and functions of cellular redox sensor HSCARG/NMRAL1, a linkage among redox status, innate immunity, DNA damage response, and cancer. Free Radic Biol Med.

[82] Dobler R, Dowling DK, Morrow EH, Reinhardt K (2018). A systematic review and meta-analysis reveals pervasive effects of germline mitochondrial replacement on components of health. Hum Reprod Update.

[83] Innocenti P, Morrow EH, Dowling DK (2011). Experimental evidence supports a sex-specific selective sieve in mitochondrial genome evolution. Science.

[84] Dowling DK, Adrian RE (2019). Challenges and prospects for testing the mother's curse hypothesis. Integr Comp Biol.

[85] Mossman JA, Tross JG, Jourjine NA, Li N, Wu Z, Rand DM (2017). Mitonuclear interactions mediate transcriptional responses to hypoxia in *Drosophila*. Mol Biol Evol.

[86] Beck EA, Thompson AC, Sharbrough J, Brud E, Llopart A (2015). Gene flow between *Drosophila yakuba* and *Drosophila santomea* in subunit V of cytochrome c oxidase: a potential case of cytonuclear cointrogression. Evolution.

[87] Han KL, Barreto FS (2021). Pervasive mitonuclear coadaptation underlies fast development in interpopulation hybrids of a marine crustacean. Genome Biol Evol.

[88] Barreto FS, Pereira RJ, Burton RS (2015). Hybrid dysfunction and physiological compensation in gene expression. Mol Biol Evol.

