# Supplementary figures and images for: Mitonuclear mismatch alters nuclear gene expression in naturally introgressed Rhinolophus bats
Source: Front Zool. 2021 Sep 6;18:42. doi: 10.1186/s12983-021-00424-x (PMC8419968; doi:10.1186/s12983-021-00424-x)

(a)

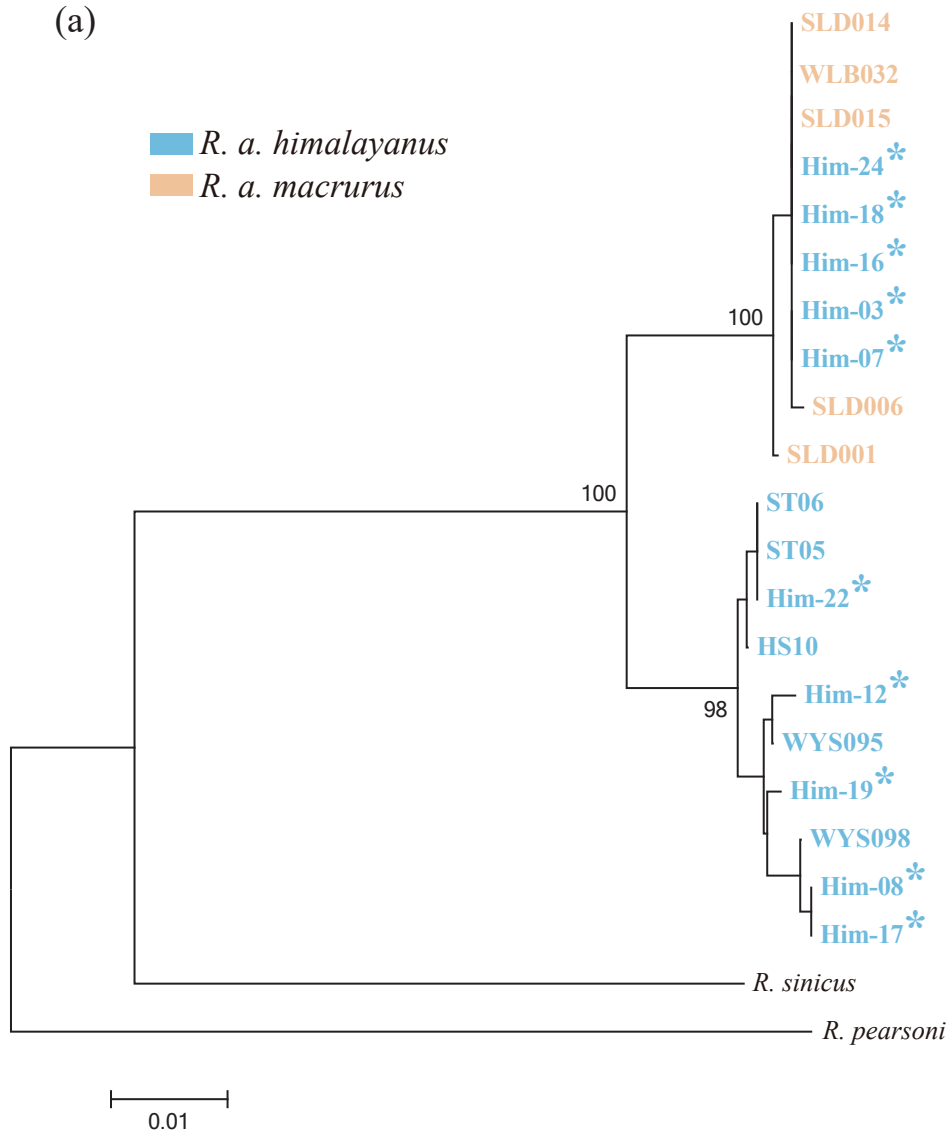

(b)

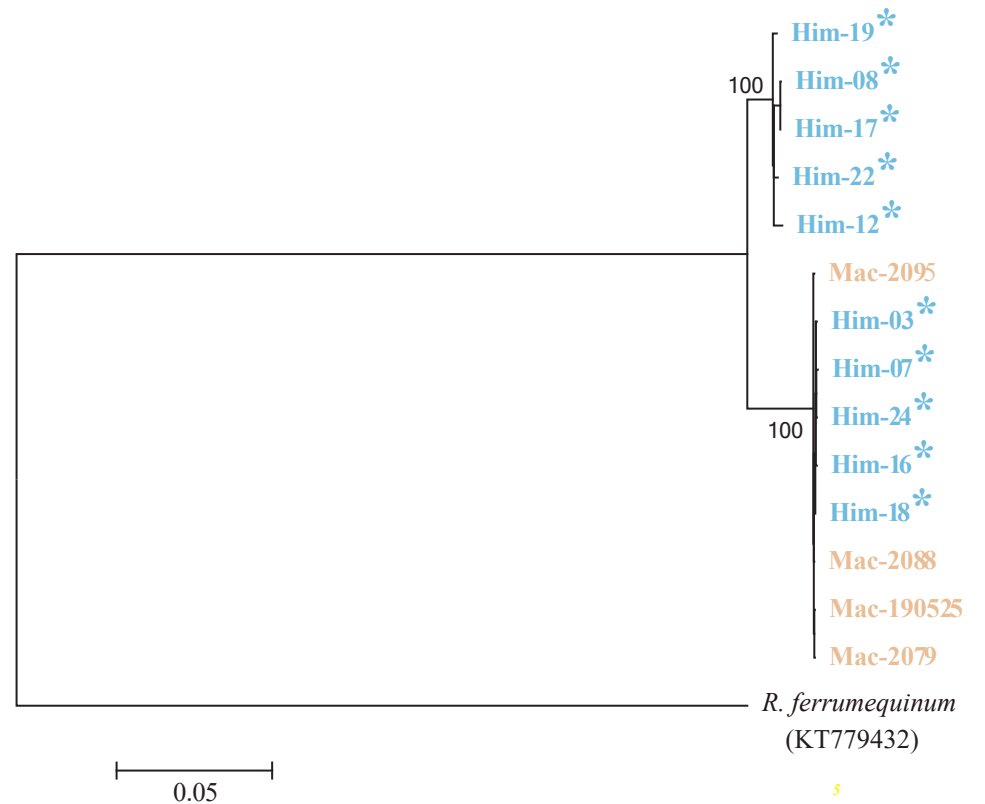

Supplement: Supplementary file 1 — Additional file 1. Phylogenetic relationships among samples of R. a. himalayanus and R. a. macrurus based on mtDNA. [file 12983_2021_424_MOESM1_ESM.pdf]

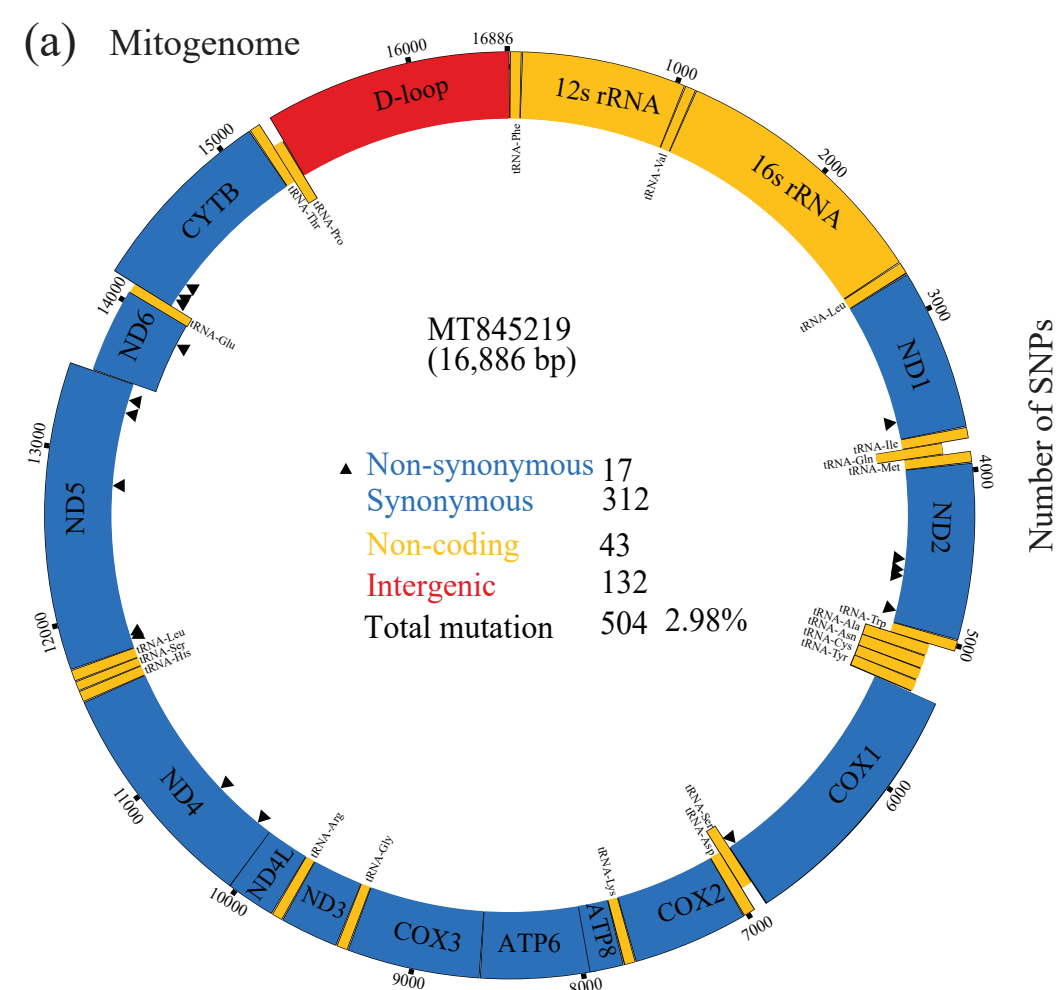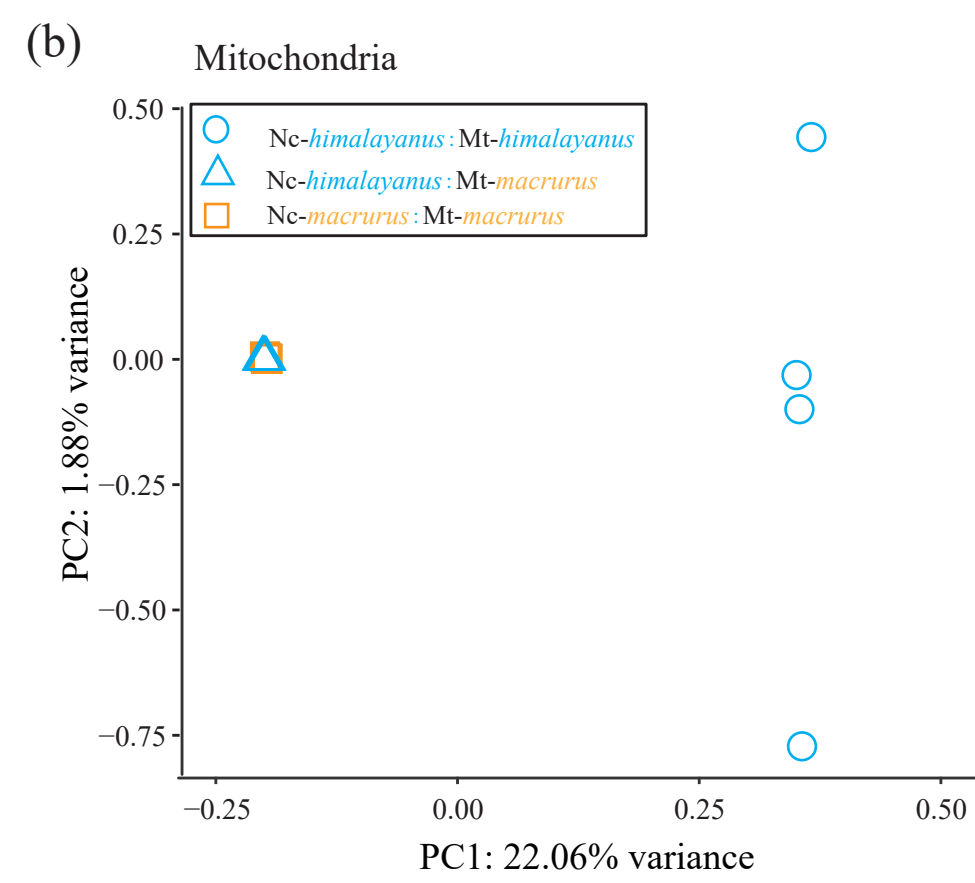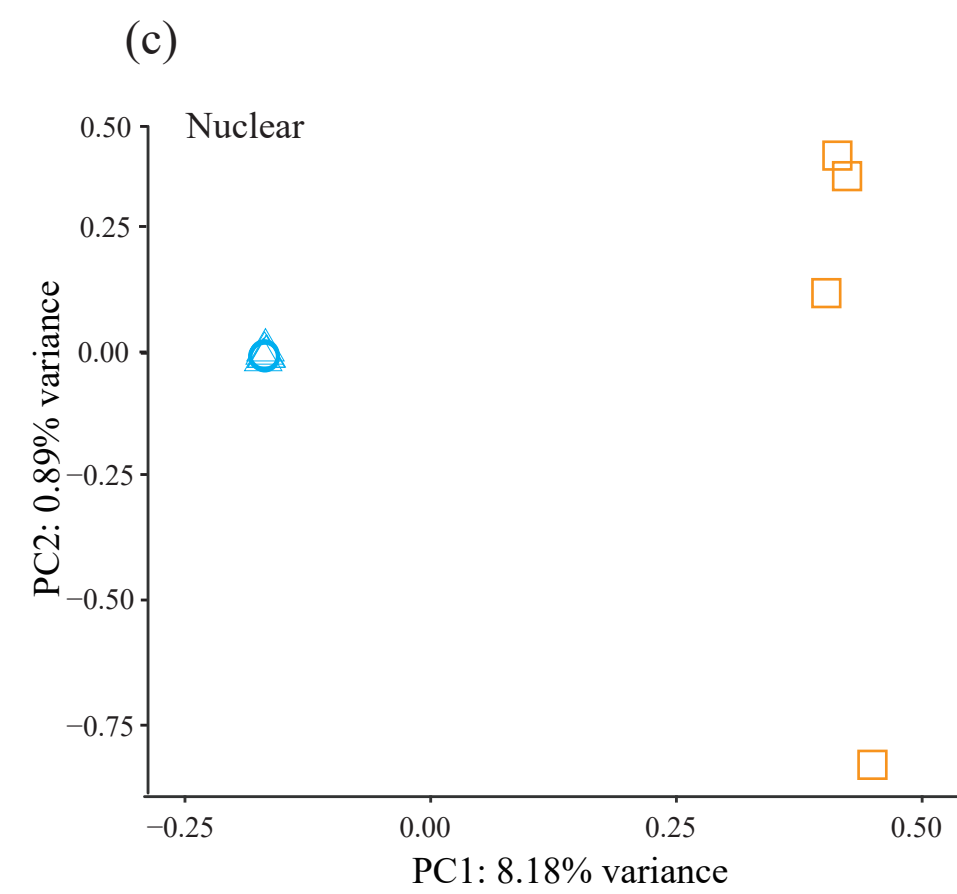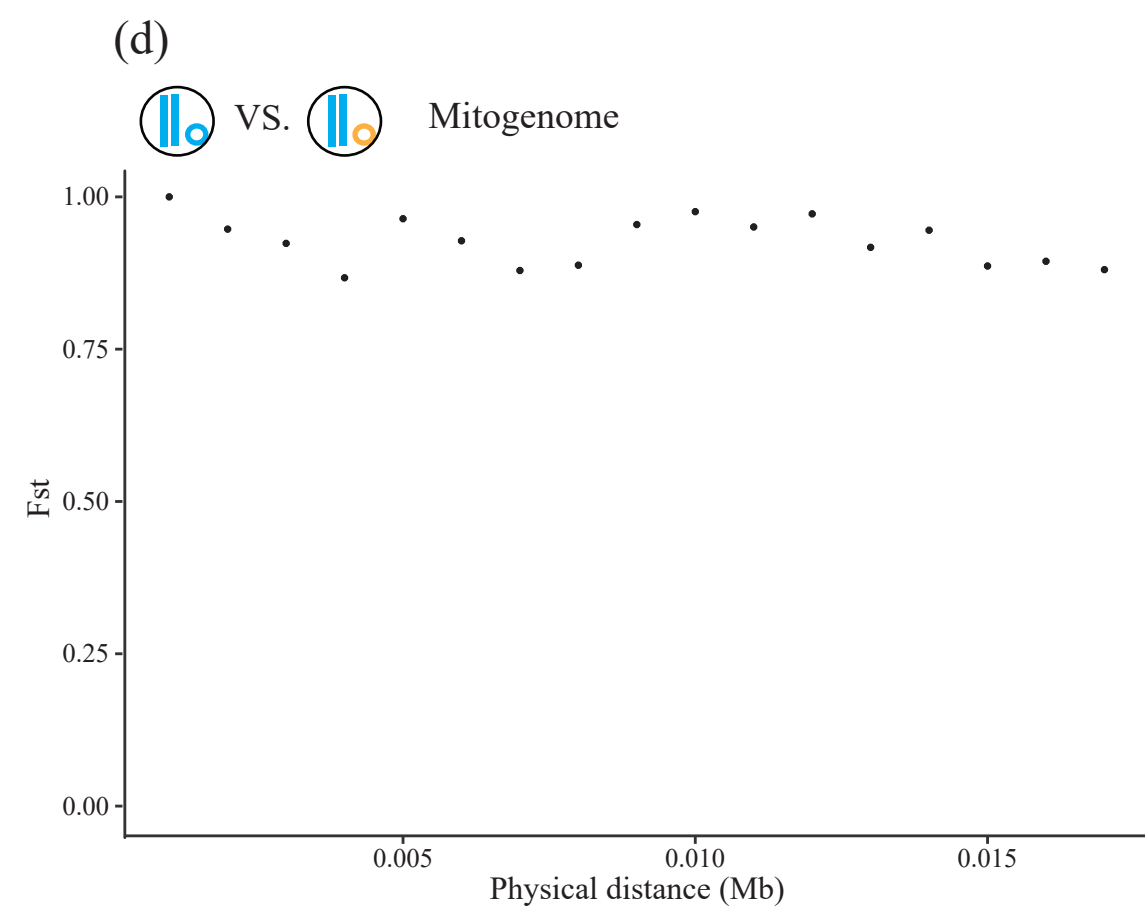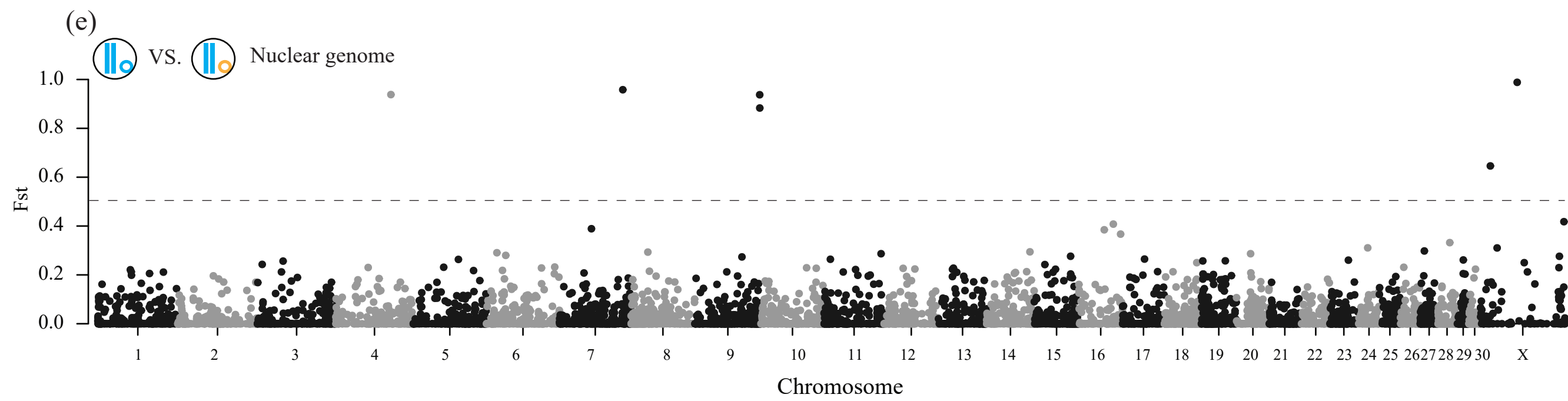

Supplement: Supplementary file 2 — Additional file 2. Sequence differences of mitogenome and nuclear genome between mitonuclear matched and mismatched individuals of R. a. himalayanus. [file 12983_2021_424_MOESM2_ESM.pdf]
