# Supplementary material for: Mitonuclear mismatch alters nuclear gene expression in naturally introgressed Rhinolophus bats
Source: Front Zool. 2021 Sep 6;18:42. doi: 10.1186/s12983-021-00424-x (PMC8419968; doi:10.1186/s12983-021-00424-x)
Supplement: Supplementary file 3 — Additional file 3. Supporting Information for “Mitonuclear mismatch alters nuclear gene expression in naturally introgressed Rhinolophus bats”. [file 12983_2021_424_MOESM3_ESM.docx]

Supporting Information for **“Mitonuclear mismatch alters nuclear gene expression in naturally introgressed *Rhinolophus* bats”**

Yuting Ding^1#^, Wenli Chen^2#^, Qianqian Li^2^, Stephen J. Rossiter^3^, Xiuguang Mao^2,4*^

1 Institute of Estuarine and Coastal Research, East China Normal University, Shanghai 200062, China

2 School of Ecological and Environmental Sciences, East China Normal University, Shanghai 200062, China

3 School of Biological and Chemical Sciences, Queen Mary University of London, London E1 4NS, UK

4 Institute of Eco-Chongming (IEC), East China Normal University, Shanghai 200062, China

# These authors contribute equally to this study

*Corresponding author: xgmao@sklec.ecnu.edu.cn

**Materials and methods**

*Generation of sequences of OXPHOS genes*

To further test for the genome similarity of the two *himalayanus* groups, we examined sequence differences at nuclear-encoded OXPHOS genes because their proteins show direct interactions with mitochondrial encoded proteins. For this, we downloaded coding sequences of 77 OXPHOS genes from four bat species generated in Shen et al. (2010) (see Table S6 for GenBank accessions). To obtain orthologous sequences of these genes in *R. affinis*, we conducted *de novo* transcriptome assembly based on all trimmed reads of the brain and muscle tissues using TRINITY (Haas et al. 2013) with default parameters. Then we performed BLASTN (the E-value < 10^-5^) searches against these assemblies using sequences of 77 OXPHOS genes as the query. This yielded sequences of 68 OXPHOS genes in *R. affinis*, which were used as the reference to obtain corresponding sequences from each of the 14 individuals (10 *himalayanus* and 4 *macrurus*). Specifically, trimmed reads of the brain tissue from each individual were mapped to the reference using BWA-MEM. SAMtools Version 1.9 was used to generate sorted BAM file and to remove potential PCR duplicates. We used BCFtools Version 1.9 to obtain consensus sequence of each gene in each individual. All sequences were then aligned using MEGA 6 and amino acid changes were calculated across the 14 individuals.

*Generation of the complete mitogenome for Him-16*

We generated the complete mitogenome for Him-16 (mismatched group) using similar procedures as in Ding et al. (2021). Specifically, we first mapped filtered reads of the genome resequencing data from this individual to the mitogenome sequence of Him-17 using BWA-MEM. SAMtools was used to generate sorted BAM files with removal of potential PCR duplicates. Mapped mitochondrial reads were retrieved and were used to assemble mitochondrial genome implemented in A5-miseq version 20160825 with default parameters (Coil et al. 2015).

*Generation of sequences of mitochondrial protein-coding genes*

To investigate whether sequence differences between Him-16 and Him-17 were fixed in each group, we generated the 13 mitochondrial protein-coding (PCGs) genes for the remaining eight *himalayanus* individuals and four *macrurus* individuals. For each individual, we used similar procedures as above to generate mitochondrial contigs based on filtered reads of RNA-seq for brain tissue. Then, sequences of the 13 PCGs genes were retrieved by performing BLASTN (the E-value < 10^-5^) using mitochondrial contigs of each individual as queries and corresponding sequences of Him-17 as references.

**Reference**

Coil D, Jospin G & Darling AE. (2015) A5-miseq: an updated pipeline to assemble microbial genomes from Illumina MiSeq data. *Bioinformatics*, 31(4), 587-589.

Haas BJ, Papanicolaou A, Yassour M, Grabherr M, Blood PD, Bowden J, Couger MB, Eccles D, Li B, Lieber M & MacManes MD. (2013) De novo transcript sequence reconstruction from RNA-seq using the Trinity platform for reference generation and analysis. *Nature protocols*, 8(8), 1494-1512.

Shen YY, Liang L, Zhu ZH, Zhou WP, Irwin DM & Zhang YP. (2010) Adaptive evolution of energy metabolism genes and the origin of flight in bats. *Proceedings of the National Academy of Sciences*, 107(19), 8666-8671.

Figure S1. Phylogenetic relationships among samples of *R. a. himalayanus* and *R. a. macrurus* based on mtDNA. (a) A Neighbor-Joining tree reconstructed based on sequences of cytochrome b gene. * indicates new *himalayanus* samples used in this study and other samples are cited from Mao et al. (2014). (b) A Maximum-likelihood tree reconstructed based on 13 concatenated mitochondrial protein-coding genes. Samples included 10 *himalayanus*, 4 *macrurus* and one *R. ferrumequinum* (GenBank KT779432).

Figure S2. Sequence differences of mitogenome and nuclear genome between mitonuclear matched and mismatched individuals of *R. a. himalayanus*. Sequence differences between matched (Him-17) and mismatched (Him-16) individuals in mitogenome (a). See also Table S2 for detailed differences in each position of mitogenome. PCAs showing memberships among all 10 *R. a. himalayanus* and 4 *R. a. macrurus* samples based on SNPs from mitochondrial (b) and nuclear (c). SNPs were generated based on RNA-seq data of the brain tissue. Sliding windows showing Fst values based on SNPs in (b and c) across mitogenome (d) and nuclear genome (e) with window sizes of 1 Kb and 100 Kb, respectively.

Table S1. Detailed sequencing information of RNA-seq data and alignment rate to the reference genome for each sample.

Table S2. Detailed differences in each position of the whole mitogenome between mitonuclear matched (Him-17) and mismatched (Him-16) individuals generated by whole-genome resequencing.

Table S3. Fourteen fixed amino acid changes between matched group (Nc-*himalayanus*:Mt-*himalayanus*) and either mismatched group (Nc-*himalayanus*:Mt-*macrurus*) or *macrurus* at mitochondrial protein coding genes.

Table S4. Fixed amino acid changes between two *himalayanus* groups (Nc-*himalayanus*:Mt-*himalayanus* and Nc-*himalayanus*:Mt-*macrurus*) and *macrurus* at nuclear-encoded OXPHOS genes.

Table S5. Significant GO terms enriched on differentially expressed genes (DEGs) identified in pectoral muscle. GO terms named in Figure 3 are shown in bold.

Table S6. List of 77 nuclear-encoded OXPHOS genes used in Shen et al. (2010).
